# The role of character goals and changes in body position in the processing of events in visual narratives

**DOI:** 10.1186/s41235-019-0176-1

**Published:** 2019-07-08

**Authors:** Ryan D. Kopatich, Daniel P. Feller, Christopher A. Kurby, Joseph P. Magliano

**Affiliations:** 1 Northern Illinois University, DeKalb, IL USA; 2 Georgia State University, Atlanta, GA USA; 3 Grand Valley State University, Allendale, MI USA

**Keywords:** Event cognition, Event segmentation, Visual narratives, Comics

## Abstract

**Background:**

A growing body of research is beginning to understand how people comprehend sequential visual narratives. However, previous work has used materials that primarily rely on visual information (i.e., they contain minimal language information). The current work seeks to address how visual and linguistic information streams are coordinated in sequential image comprehension. In experiment 1, participants viewed picture stories and engaged in an event segmentation task. The extent to which critical points in the narrative depicted situational continuity of character goals and continuity in bodily position was manipulated. The likelihood of perceiving an event boundary and viewing latencies at critical locations were measured. Experiment 1 was replicated in the second experiment, without the segmentation task. That is, participants read the picture stories without deciding where the event boundaries occurred.

**Results:**

Experiment 1 indicated that changes in character goals were associated with an increased likelihood of segmenting at the critical point, but changes in bodily position were not. A follow-up analysis, however, revealed that over the course of the entire story, changes in body position were a significant predictor of event segmentation. Viewing time, however, was affected by both goal and body position shifts. Experiment 2 corroborated the finding that viewing time was affected by changes in goals and body positions.

**Conclusion:**

The current study shows that changes in body position influence a viewer’s perception of event structure and event processing. This fits into a growing body of research that attempts to understand how consumers of multimodal media coordinate multiple information streams. The current study underscores the need for the systematic study of the visual, perceptual, and comprehension processes that occur during visual narrative understanding.

## Significance

Sequential visual narratives (comics and picture stories) are a prevalent way that we encounter narrative experiences. Despite this fact, until relatively recently, this is a medium that has received little attention in the psychological sciences. One aspect of the sequential visual narratives of interest in the present study is that they are multimodal in nature. Specifically, they contain images and text that convey the narrative events. While practitioners have speculated as to how these information sources are coordinated to convey a coherent narrative experience, there has been little empirical work on how images and text are processed in conjunction. The goal of the present study was to explore the relative contributions of text and images in conveying the event structure of a sequential visual narrative. Our study shows that verbal content that conveys the goals of characters and visual content that depicts changes in the bodily positions of characters (e.g., conveying different actions) independently impact a viewer’s understanding of the narrative events. The results of this study make unique contributions to the growing research on visual narrative comprehension, traditional research on text comprehension, and the practice of creating sequential visual narratives.

## The role of character goals and changes in body position in the processing of events in visual narratives

Visual narratives are a ubiquitous modern experience that can occur as static images with accompanying text (e.g., picture books, comics), or as continuous visual experiences that incorporate aural stimuli (e.g., film, TV). Most visual narratives are multi-modal in nature in that they contain visual and linguistic content. As a result, when engaging with visual narratives, consumers must integrate across these modalities in order to understand them (Cohn, 2016; Magliano, Loschky, Clinton, & Larson, 2013). While there is growing interest in the study of visual narrative comprehension, many researchers have opted to use materials that do not contain language (e.g., Magliano, Kopp, McNerney, Radvansky, & Zacks, 2012; Magliano, Larson, Higgs, & Loschky, 2016; Magliano & Zacks, 2011; Zacks, Speer, & Reynolds, 2009; Zacks, Speer, Swallow, & Maley, 2010). There is some evidence, however, that both visual and linguistic information support comprehension (e.g., Magliano, Dijkstra, & Zwaan, 1996). Moreover, there is robust evidence that visual and linguistic content support learning from multimedia contexts (e.g., Mayer, 2009), and there is reason to believe that visual and linguistic content support narrative comprehension in profound ways. However, because these information streams are not always equally important in conveying a narrative (e.g., Cohn, 2016), how visual and linguistic information convey meaning is an open question. In the present study, we explored the extent to which visual and linguistic content in sequential visual narratives (i.e., visual narratives that consist of static images such as comics) support comprehension and in what context these sources of information convey important and distinct information about the story.

Theories of text comprehension universally assume that narrative comprehension requires one to construct a coherent mental model that, in part, reflects how the explicitly conveyed narrative events are situationally related (e.g., related in terms of space, time, causality; Zwaan, Magliano, & Graesser, 1995 & Zwaan & Radvansky, 1998). Narrative plots are structured around characters performing intentional actions (Gee & Kegl, 1983; Mandler & Johnson, 1977; Rumelhart, 1975; Stein & Nezworski, 1978; Trabasso, van den Broek, & Suh, 1989). As such, inferring the relationships between explicitly conveyed actions and the goals that motivated them is an important basis for constructing coherent mental models (Long, Golding, & Graesser, 1992; Magliano, Taylor, & Kim, 2005; McNamara & Magliano, 2009; Suh & Trabasso, 1993). Research has shown that these mental models are organized in terms of events and event structure, with distinct boundaries between them (e.g., Kurby & Zacks, 2008; Radvansky, 2012; Radvansky, Krawietz, & Tamplin, 2011; Radvansky & Zacks, 2014). Comprehenders tend to track a set of situational dimensions across a narrative and segment their representation when these dimensions change. For example, people will track, from moment to moment, the goal of a character. If that goal changes in the currently processed information unit (e.g., sentence, comic panel, movie scene, etc.), people tend to update their mental models to accommodate that change (Kurby & Zacks, 2012; Zacks & Swallow, 2007; Zwaan, Langston, & Graesser, 1995), and such updating leads to the perception of an event boundary (Speer, Zacks, & Reynolds, 2007). This additional processing at event boundaries confers behavioral and cognitive consequences: reading times tend to slow down at changes (Radvansky & Copeland, 2010; Zacks, Kumar, Abrams, & Mehta, 2009; Zwaan, Magliano, & Graesser, 1995), memory increases for the new content (Swallow, Zacks, & Abrams, 2009), and memory for previous event information becomes less accessible than current event information (Radvansky et al., 2011; Speer & Zacks, 2005; Swallow et al., 2009). In the current study, given that goal changes are predictive of event segmentation and updating, we ask how might viewers of sequential narratives extract goal information during event processing?

**Fig. 1:**
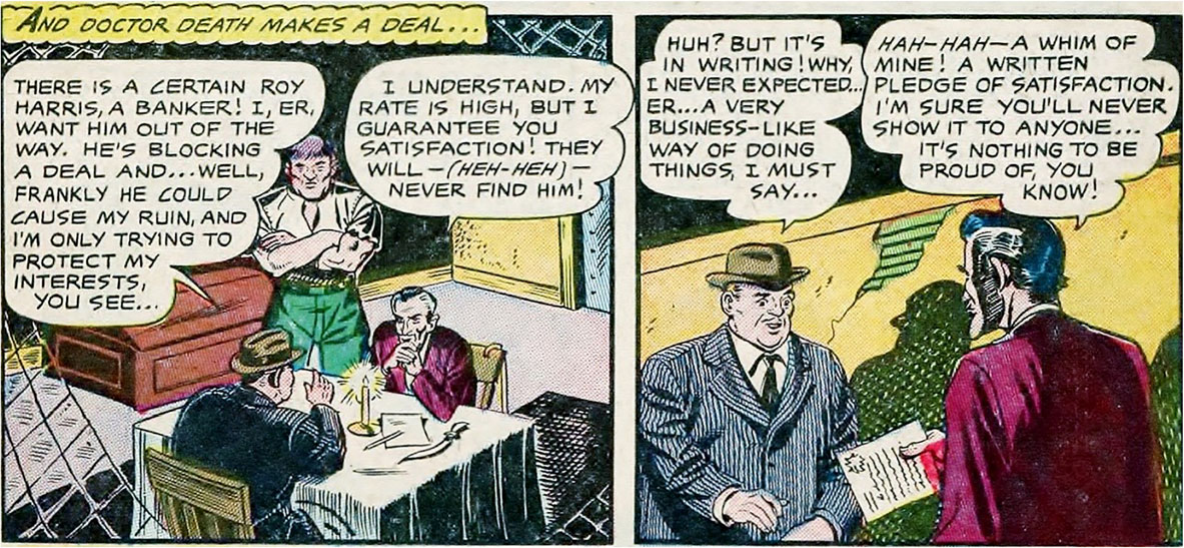
Two panels from the graphic story Black Cobra (1954). In these panels, the bodily positions convey consistency with the stated goal (i.e., the client is given a contract from the hit man). Available from https://digitalcomicmuseum.com/index.php?cid=735

Visual and linguistic content in picture stories can be coordinated to understand how the actions of the characters are related to explicitly established goals. Sometimes these information streams indicate that there is continuity of goal structure. For example, consider the two-panel sequence in Fig. 1 from the comic Black Cobra (1954). In the first panel, two men are seated at a table. The dialogue between the characters establishes that one character has the goal of having an individual killed, and the other character is a hit man who can accomplish that goal. Their bodily positions are consistent with that goal in that they convey that the characters are having a conversation about the contract. The language and images in the second panel are coherently related to the first panel because they are consistent with the continuation of the goal. Specifically, the dialogue conveys that the hit man has agreed and has established a written contract, and the bodily positions of the characters also convey that the contract has been accepted (i.e., the hit man is handing a piece of paper to the client). Presumably, a mental model of the events in the two panels would reflect the situational consistencies between the language and the visual content in terms of the goals of the characters. We see the situation reflected in this panel as akin to situations in text in which a reader has to infer how an explicitly described action is causally related to an explicitly established goal (e.g., Suh & Trabasso, 1993). However, in this example the action and goal are in the same panel.

**Fig. 2:**
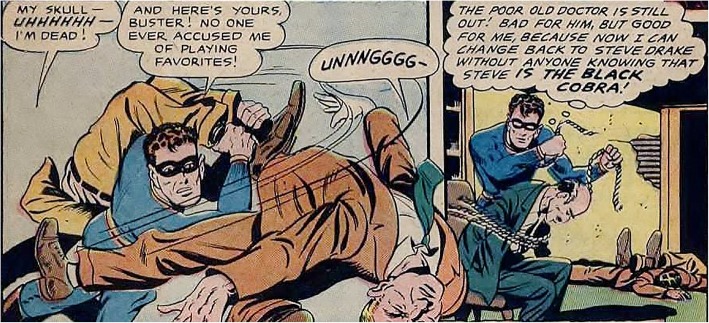
Two panels from the graphic story Black Cobra (1954). In these panels, the bodily positions in the second panel convey that a change in goals has occurred (i.e., the Black Cobra is rescuing the doctor rather than fighting his enemies). Available from https://digitalcomicmuseum.com/index.php?cid=735

However, visual and linguistic information streams may also indicate shifts in goals of characters. For example, consider the two-panel sequence in Fig. 2 from the same comic. In the first panel, the dialogue implies that the characters are in the middle of a fight and the visual stream gives specific details on who is fighting and how the fight is progressing. Specifically, the Black Cobra is using one of his enemies’ bodies to attack another. The second panel takes place in the same location - indicated by the Black Cobra’s enemies in the background - and involves the Black Cobra rescuing a doctor. Importantly, the bodily positions and actions of characters have dramatically changed and reflect that their goals have changed (i.e., rescuing someone is a separate goal from fighting one’s enemies). The introduction of a new character, actions of the primary characters (i.e., the Black Cobra), and the dialogue all convey that there is a shift in the goals of the primary character.

Figure 1 reflects the situation that we were interested in investigating because it resembles a common situation in narrative texts but has features unique to visual narratives. Specifically, as discussed above, text specifies content that conveys a goal of the characters (e.g., the statement of guaranteeing satisfaction indicates that the job has been accepted) and the action conveyed can be understood as being in the service of accomplishing that goal (e.g., handing over a contract). Viewers of visual narratives similarly need to infer the relationships between character actions and goals, but those actions are conveyed in pictures via changes in bodily position from panel to panel (e.g., the hit man was previously sitting at a table, clasping his hands). As such, inferring the relationship between texts conveying goals and visually depicted actions necessarily requires an integration of linguistic and visual content. Given that the study of visual narratives is relatively new, this phenomenon has not received empirical attention. Moreover, given the importance of inferring the relationships between goals and actions in establishing narrative coherence (e.g., Suh & Trabasso, 1993), this is a candidate for exploring how verbal and visual content are processed and integrated to support visual narrative comprehension.

Linguistic and visual content can have different relationships in conveying the narrative. Cohn (2016) argues that information streams in multimodal narratives vary along two dimensions: dominance and assertiveness. Dominance refers to the degree to which an information stream carries semantic information that contributes to constructing a mental model of the narrative (Cohn, 2016). For example, Figs. 1 and 2 both show co-dominant visual and verbal information streams in which both the pictures and the dialogue contribute to the understanding of the narrative. In addition to dominance, multimodal narratives may also vary in their assertiveness, or the degree to which there is a grammar-like structure that dictates order (Cohn, 2016). In the first panel of Fig. 2, the Black Cobra is shown fighting his enemies. This panel is followed by an image of him rescuing the doctor, with his enemies incapacitated in the background. Thus, the order of pictures clearly cannot be switched without changing the understanding of the narrative. On the other hand, in Fig. 1, if the pictures were reversed, the meaning of the visual sequence would not be changed (the characters sitting at the table in conversation and exchanging the contract could visually happen in any order). In both Figs. 1 and 2, we can see that the order of the text cannot be changed without altering the narrative. Thus, Fig. 1 would be described as a co-dominant, verbal-assertive narrative, whereas Fig. 2, would be described as a co-dominant, co-assertive narrative (Cohn, 2016). Although there any many relationships between verbal and visual streams in visual narratives, for the purpose of the current study, we focused on co-dominant narratives, with co-assertive relationships around the critical point. Specifically, we were interested in seeing whether changes in body position and changes in goal continuity were processed independently when visual and verbal information streams are co-dominant as well as co-assertive.

### Overview of study, hypothesis, and predictions

The goal of the present study was to explore the relative impact of the linguistic and visual content on the processing of sequential narratives. We explored this issue in the context of event segmentation. People habitually recognize the boundaries that make up mundane everyday events (Speer, Swallow, & Zacks, 2003; Zacks, Speer, Vettel, & Jacoby, 2006; Zacks, Tversky, & Iyer, 2001) and narratives (Kurby & Zacks, 2012; Magliano et al., 2012; Magliano, Miller, & Zwaan, 2001; Magliano et al., 2005; Zacks, Speer, Swallow, Braver, & Reynolds, 2007). Segmentation for mundane, everyday activities is heavily influenced by perceptually salient changes in bodily position (Zacks, 2004), presumably because these are informative of progression towards the accomplishment of a goal (Kurby & Zacks, 2011). In the context of narratives, segmentation is influenced by changes in situational continuities (time, space, causality, goals) such that boundaries between narrative events are perceived when there are shifts in these dimensions (Kurby & Zacks, 2012; Magliano et al., 2012, 2001, 2005; Zacks, Kumar, et al., 2009). Importantly, when the visual stream suggests that there are changes in the goals of characters in the context of picture stories (Magliano et al., 2012) or films (Magliano et al., 2005; Magliano & Zacks, 2011; Zacks, Kumar, et al., 2009), viewers tend to perceive a narrative boundary.

**Fig. 3:**
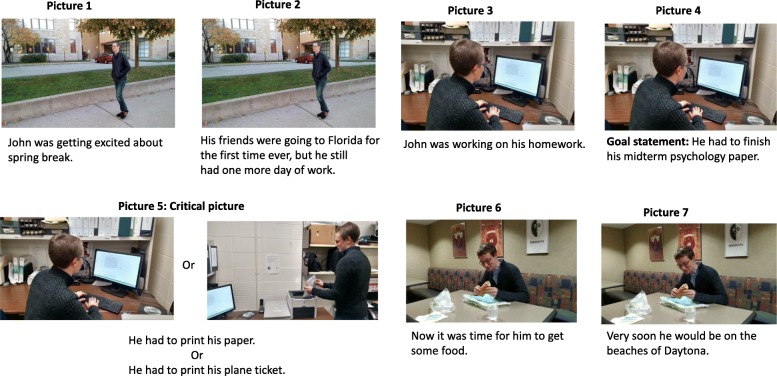
An example critical story. In this particular example, the goal of completing homework is maintained, but the bodily position is different than in the previous panel. This slide could also contain text that establishes a new goal (e.g., the character had to print his plane ticket), the picture from slides 3 and 4, or any combination thereof (see Fig. 4 for possible conditions)

**Fig. 4:**
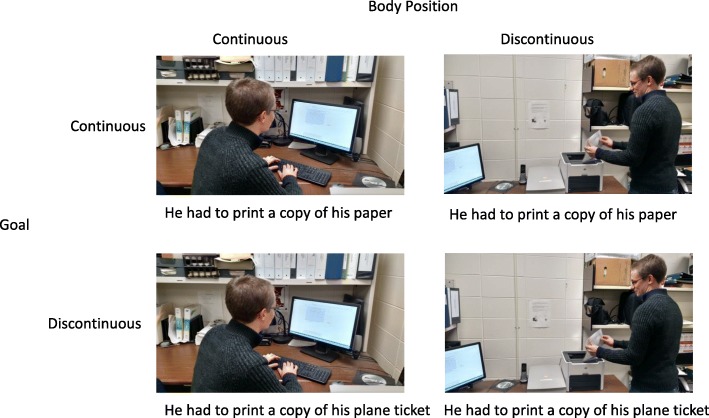
Example critical slide in the four conditions of the 2 (Goal: Continuous and discontinuous) × 2 (Body position: Continuous and discontinuous) design

In the first experiment of this study, participants viewed sequential narratives that contained text (see Fig. 3). The text provided a narration of the events depicted in the pictures, and the pictures depicted the characters engaged in goal-directed activities. An explicit goal (e.g., finish psychology paper) of the character was established in the verbal content (see first and second panels in Fig. 3). At a critical panel (panel 5 in Fig. 3), we manipulated the extent to which that the verbal content conveyed an event that was either continuous (e.g., needed to print the paper) or discontinuous (e.g., needed to print a plane ticket) with the prior established goal (see Fig. 4 for a representation of the four conditions of the experiment). We also manipulated whether the bodily position in the critical panel was continuous with that of the prior panel or indicated a change in position (i.e., discontinuous). Importantly, the changes in bodily position could be interpreted as being either consistent or inconsistent with the prior goal (e.g., the character could be printing the paper or printing the plane ticket). In experiments 1 and 2, participants read the pictured stories. In experiment 1, we recorded both viewing time and segmentation behavior via a unitization task, and in experiment 2 we recorded viewing time only. The unitization task involved participants indicating when there were changes in the events conveyed in the story pictures. Participants were not instructed about what constitutes a change in events, rather, they were allowed to determine this with no experimenter input. Segmentation judgments have been found to be highly reliable within and across participants (Magliano et al., 2012; Zacks et al., 2001). Additionally, these judgments are correlated with theoretically meaningful changes in videos (Zacks, Kumar, et al., 2009) and text (Speer et al., 2007; Speer & Zacks, 2005; Zacks, Kumar, et al., 2009).

There are two possible hypotheses for how people process changes in goal status and bodily positions when comprehending static visual narratives. First, it may be that changes in body position and goals are processed independent of each other. If this is the case, the probability of segmentation would increase when there is a shift in goal and when there is a shift in body position, but there would be no interaction. Alternatively, it is possible that there is an interaction between goal and body position such that shifts in body position increase segmentation likelihood, but only when goal continuity is maintained. Specifically, the disruption of the goal in the discontinuous goal condition will overshadow the impact of the body shift in the body discontinuous condition. However, in the continuous goal condition, the shift in bodily position indicates incremental and meaningful change toward the completion of the goal (e.g., completing the paper) that may require updating (Kurby & Zacks, 2012).

## Experiment 1

### Method

#### Participants

A total of 91 undergraduate students (35 female; mean age = 19.89, SD = 3.31) at a large Midwestern university completed the study. Participants were given course credit in exchange for participation. Two participants’ data were deleted due to computer malfunction and noncompliance. Trial viewing times were examined after these participants’ data were deleted. Outliers were removed based on viewing times for the critical slide for either being too quick (less than 500 ms; Zacks, Kumar, et al., 2009) or for being too slow (greater than 3 SD above the mean) at the item level. This resulted in all data from an additional participant being eliminated from the analyses. Thus, the final sample comprised 88 participants. In total, data cleaning resulted in a loss of 4.0% of items.

#### Design

The study employed a 2 (Goal continuity: continuous vs. discontinuous) × 2 (Bodily position: continuous versus discontinuous) repeated measures design. There were two dependent measures. The first was segmentation behavior. For each critical item, participants made a dichotomous decision indicating whether or not the panel constituted an event boundary. The second dependent measure was picture viewing time, which was defined as the length of time between the onset of a picture on the computer screen and the pressing of the next button to advance the picture. Picture viewing times were recorded in millisecond accuracy.

#### Materials

A series of 32 seven-panel stories about day-to-day events in a college couple’s life were constructed for participants to read and segment. Of these, 16 were critical stories, which contained an identical structure (see Fig. 3 for an example). The first two panels contained text that established a global goal for the narrative (e.g., the character had to complete coursework before spring break) and contained a picture (e.g., the character was walking through campus). In the third and fourth panel, a sub goal of the larger goal was established via the text (e.g., the character was writing a paper for a psychology course) and a new picture was shown (e.g., the character sitting at the computer screen). This picture showed behavior consistent with that sub goal. In the fifth “critical” panel, the text either conveyed a continuation of the current goal (e.g., the character was printing the paper) or was discontinuous with the current goal (e.g., the character remembered he had to print his plane ticket), and the picture was either the same as the one presented in the third and fourth panels or a new picture was presented with the character in the same location, but in a different body orientation (e.g., the character was standing next to the printer). The goal discontinuous condition involved events that disrupted the current goal such that it was no longer active at the critical slide. The change in bodily position was such that it could be an action that was causally related to the established goal (e.g., the goal to finish the paper) or the disruption (e.g., the need to print the plane ticket). However, within a given version of a story, the change of bodily position was most causally related to text presented in the critical slide (e.g., printing the paper or a plane ticket). Specifically, the co-activation of the pictures and texts in working memory at the critical items lead to them being causally connected (Fletcher & Bloom, 1988). Text in the critical panel did not differ in number of syllables between conditions (*p* > .10). The sixth and seventh panels conclude the narrative and contain a different picture from the previous panels and text that indicated that the prior goals were completed (e.g., the character is in the cafeteria having dinner after having finished his work). Figure 4 shows an example of critical panels for each condition of the 2 × 2 design. Participants saw only one version of each story. Stories were assigned to conditions via a 4 × 4 Latin square, which yielded four counterbalanced schemes. There were four critical items per cell for each counterbalancing scheme. Participants were randomly assigned to one of these counterbalanced schemes.

In addition to the 16 critical stories, 16 filler stories were created to mask the structure of the critical items. These filler narratives varied in the number of pictures and goals that were presented. Of these, eight stories were constructed to create an overarching narrative that followed the course of the main characters’ relationship. This was done to make the task more engaging for participants. The filler stories conveyed the story of the two characters’ courtship and eventual decision to end their romantic relationship. The events of the critical stories were such that they could occur in any order, but the eight filler items that conveyed the story had to occur in a fixed order to convey the progression of the story (i.e., decision to date, period in which they dated, the breakup). As such, those filler stories were presented in a fixed order (i.e., the 1st, 2nd, 9th, 10th, 17th, 24th, 25th, and 32nd items were fixed) and the order of presentation of the critical items and the remainder of the filler stories was randomized for each participant. Experimental and filler stories can be viewed and downloaded (https://osf.io/b4drz/).

#### Procedure

Participants were greeted, completed informed consent forms, and asked to answer two demographic questions on a sheet of paper (i.e., sex and age). They were instructed that they would read a series of stories about college students, and that there was an overarching story that was conveyed over several episodes. They were instructed that experimenters were interested in learning how people make sense of the events in stories. To accomplish this, they were to read the stories, slide by slide, and identify when they felt that there was a meaningful change in events, such that one event ended or another had begun. Participants were told that there was no right or wrong way of doing this activity and that it was up to them to determine what a meaningful change in events was. After receiving instructions, participants completed a practice item that had the same structure as the non-story filler items. Next, they were instructed to read the critical and filler items as described above. These were presented one slide at a time on a computer screen. Pictures and texts were shown concurrently with text displayed directly below each picture. All text was left justified to the edge of the picture. Participants pressed the enter key to progress from slide to slide and were instructed to press the spacebar instead whenever they felt that one meaningful event had ended or another had begun. All stimuli, including the practice item, were presented using E-Prime version 2.0 (Psychology Software Tools, 2011). Participants were instructed to keep their hands on the keyboard at all times and were monitored to make sure that they followed that instruction. Finally, participants were debriefed and thanked for their time.

## Results and discussion

**Fig. 5:**
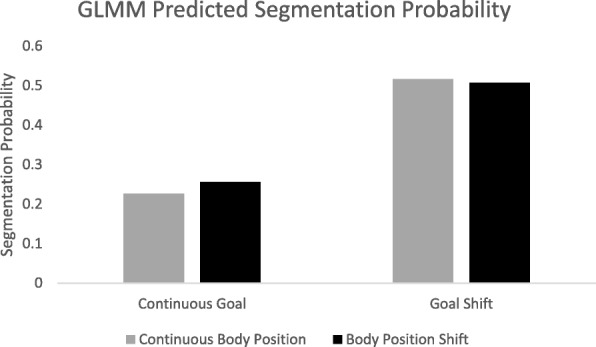
Predicted segmentation likelihood from logistic mixed effects model by condition. GLMM, generalized linear mixed model

All analyses were performed using the lme4 package in R (Bates et al., 2015). As our primary dependent measure was the likelihood of segmentation, we first fitted a null logistic mixed effect model with segmentation at the item level as the outcome variable. Item and subject were treated as random effects. This analysis revealed that significant variance in segmentation was accounted for by subject (intraclass correlation coefficient (ICC) = .39), but not by item (ICC = .02), but we kept both in the model regardless, to better account for random factor variability (Barr, Levy, Scheepers, & Tily, 2013). Next, the fixed effects of goal shift, change in body position, and the interaction between goal shift and change in body position terms were added as predictors of segmentation. Neither change in body position nor the interaction between goal shift and change in body position were significant predictors of segmentation likelihood. The model coefficients are presented in Table 1 and predicted values from the model are displayed in Fig. 5. The data suggest that when a goal shift occurred, the odds of segmenting at the critical slide were 3.53 times as large as when the goal was maintained.

**Table 1:** Model estimates for logistic linear mixed model predicting segmentation

	Coefficient	SE	*z*	*p*
Fixed effects (log transformed)				
Intercept	−1.22	0.24	−5.14	< .001
Goal	1.26	0.20	6.47	< .001
Body position	0.14	0.20	0.71	.481
Goal × Body Position	−0.19	0.27	−0.69	.489
Random effects				
Subject	2.44	1.56		
Item	0.12	0.35

**Fig. 6:**
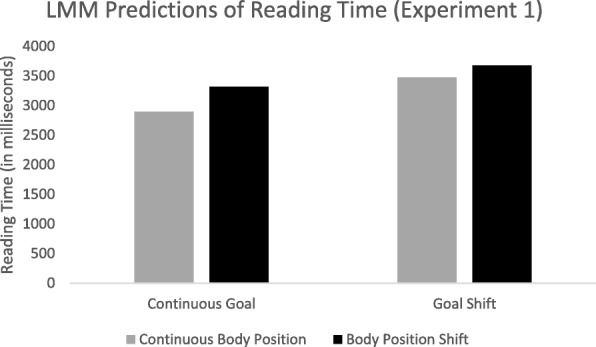
Predicted reading times for experiment 1 from linear mixed effects model by condition. LMM, linear mixed model

In addition to segmentation, separate linear mixed models were constructed using viewing time as the outcome variable. Item and subject were treated as random in the null model. Both item (ICC = .14) and subject (ICC = .17) accounted for significant variance in viewing times. Next, the fixed effects of goal shift, change in body position, and the interaction between goal shift and body position were added. The model coefficients are presented in Table 2 and predicted reading times are presented in Fig. 6. The data suggest that shifts in goal and body position independently increased the time taken to view the critical slide (460 ms and 370 ms respectively).

**Table 2:** Model estimates for linear mixed model predicting viewing time (experiment 1)

	Coefficient	SE	df	*t*	*p*
Fixed effects (log transformed)					
Intercept	2812	201	27.5	14.02	< .001
Goal	460	111	1244.4	4.15	< .001
Body position	370	110	1243.9	3.35	< .001
Goal × Body Position	− 160	157	1244.4	−1.02	.308
Random effects					
Subject	545,903	739			
Item	448,101	669

Our primary outcome of interest was segmentation at the critical slide. We found that when a goal was shifted, participants were more likely to segment, a finding that is in line with previous research on goals in visual narratives (e.g., Magliano et al., 2005; Magliano & Zacks, 2011; Zacks, Kumar, et al., 2009). Changes in body position, however, did not increase segmentation likelihood, suggesting that participants relied on explicit changes in goals to make segmentation decisions, rather than motions related to the goal. The viewing time data suggest that both goal and body position changes affect how long participants viewed the critical pictures, with longer viewing times occurring in the discontinuous conditions for both the goal and body position variables.

### Story-wide analysis of situational features

Although our initial interest was in assessing how the critical slide was processed as a function of goal status and changes in bodily position, focusing on a single trial per story may have constrained variability associated with body position changes and event processing, reducing the likelihood of our analyses detecting an effect. The body positions changed at non-repeated pictures across the entire story. As such, to provide a more powerful test of the effect of body position shifts on behavior, we conducted a larger-scale analysis assessing the impact of changes in bodily position on segmentation, and viewing time, for the entire set of stories, across all slides of each story. We coded each slide of both the experimental and filler stories regarding whether it conveyed a change in the bodily positions of the characters relative to the prior slide. Additionally, we developed some control variables known to covary with event processing behavior. It has been documented that segmentation decisions in the context of stories are correlated with shifts in situational continuity (Magliano et al., 2012, 2001; Zacks, Kumar, et al., 2009). Two important features of the situation that are tracked and updated by readers are the causality of the events and changes in the spatiotemporal framework (Magliano et al., 2012; Trabasso et al., 1989; Zwaan & Radvansky, 1998).

The spatiotemporal framework involves the spatial locations and time frames in which the narrative events occur. While changes in space and time can be coded separately for longer narratives (e.g., Magliano et al., 2001), shifts in time and space almost always co-occurred in the present stories, and therefore were treated as one variable. A spatiotemporal shift occurred when characters changed spatial locations, which would take time to happen in the story world (e.g., picture 6 shows the character in a cafeteria, whereas he was in an office in picture 5). Shifts in the spatiotemporal framework were conveyed via visual content of the stories. Causal shifts occurred when the story events did not have necessary and sufficient causal antecedents in the immediate story episode (Trabasso et al., 1989). This happened when characters engaged in behaviors associated with a goal that was not previously established or when they experienced unexplained events. For example, in the example story in Fig. 3, John needing to print his plane ticket in picture 5 did not have a direct causal antecedent in the prior story context, was associated with a new goal, and as such, constituted causal shift. Another example causal shift was his getting food in picture 6, because this reflected a change in goal that did not have a causal antecedent in the prior story context. As a final example, in another story, a character gets an unexpected phone call, which is an example of an unexplained event that was coded as indicating a causal shift. The linguistic content was primarily used to identify the presence of causal shifts. Table 3 shows the correlation between these variables. These variables were then used to predict the segmentation and viewing time data for all pictures in the stories.

**Table 3:** Correlation matrix for situational analysis variables

	1.	2.
1. Causal Shift	–	–
2. Spatial-Temporal Shift	.39	–
3. Body Position Shift	.41	.79

### Segmentation behavior

We computed a logistic mixed effects model predicting per-slide segmentation probability with causal shifts, spatiotemporal shifts, and body position shifts as fixed effects, and subject and story as random effects. We excluded the first slide of each story from the analysis because the first slide always conveyed a new event, new spatiotemporal framework, and body positions; including them in the analysis could inflate the assessment of their impact on the dependent measures. The results are presented in Table 4. As can be seen in Table 4, all three variables significantly predicted segmentation behavior. A change on any dimension was related to an increased probability of segmentation, including a change in body position,^1^ which is consistent with the results of Magliano et al. (2012). Notably, a change in body position was associated with a significant increase in the probability of segmenting (also see Footnote 1). These results are also consistent with theoretical perspectives that assume understanders routinely monitor situational continuity to support mental model construction while processing narratives (Gernsbacher, 1990; Radvansky & Zacks, 2014; Zwaan & Radvansky, 1998) and that perceived changes in these dimensions affects the perception of event boundaries in narratives (e.g., Magliano et al., 2012; Zacks, Kumar, et al., 2009).

**Table 4:** Results from logistic mixed effects model using situational analysis variables

	Coefficient	SE	*z*	*p*
Intercept	−1.14	0.14	−7.90	<.001
Causal shift	0.51	0.04	12.15	<.001
Spatial-temporal shift	0.28	0.06	4.96	<.001
Body position shift	0.84	0.05	16.21	<.001

A change in body position was a significant predictor of viewing time when we considered the entire story. These data suggest that results for the target slides must be interpreted in the context of the fact that changes in bodily positions were either consistent with explicitly established goals (i.e., in the goal continuous condition) prior to the target pictures or the interruption event (i.e., goal discontinuous) specified in the target slides. In the latter situation, the event conveyed in the text overrode the impact of changes in bodily position on segmentation. However, in the larger story context, changes in bodily position were not necessarily associated with an explicit goal. In fact, they may have indicated that there was a new goal that needed to be inferred. Readers routinely infer goals that explain the actions of characters (Long et al., 1992), and there is reason to believe that viewers do so as well when they perceive meaningful changes in the body positions of the characters.

### Viewing time

We computed a linear mixed effect model with the same fixed and random effects as the segmentation analysis on the per-slide viewing times. Similar to the analysis above, we excluded the first slide. Additionally, we removed viewing times longer than 3 SD from the mean (cutoff 10,283 ms), and response times shorter than 500 ms, resulting in the removal of 3.96% of the trials. The results are presented in Table 5. As can be seen in Table 5, shifts in causality and a change in body position were both significantly associated with an increase in viewing time. The fact that the spatiotemporal shift predictor was not significant is anomalous given the literature on sentence reading times. It is well-established that sentence reading time tends to increase when there are shifts in causality, time, and space (e.g., Magliano, Zwaan, & Graesser, 1999; Zwaan, Magliano, & Graesser, 1995). However, in this experiment, concurrent with viewing the slides, participants made segmentation judgments, and as such making those judgments could have masked the impact of situational shifts or changed the relationship between situational changes and viewing time. Nonetheless, changes in bodily position were a significant predictor of processing time beyond the target pictures.

**Table 5:** Results from linear mixed effects model using situational analysis variables (experiment 1)

	Coefficient	SE	df	*t*	*p*
Intercept	2922.52	131.96	65.82	22.15	<.001
Causal shift	417.23	28.47	16,500.91	14.66	<.001
Spatial-temporal shift	9.13	39.64	16,496.17	0.23	.818
Body position shift	271.53	35.53	16,501.63	7.64	<.001

This finding is consistent with work by Hard et al. (2011). Hard et al. (2011) had participants watch slideshows of everyday actions and similarly showed that changes in bodily position that implied an update in the action sequence led to an increase in processing time. This indicates that both semantic information that conveys the continuity or discontinuity of goals and changes in bodily position affect the moment to moment processing of visual narratives. Experiment 2 of the current study was designed to explore these effects outside the context of the segmentation task.

## Experiment 2

As noted above, the viewing time data for experiment 1 may have been affected by the fact that participants were also making segmentation judgments. As such, we conducted experiment 2 such that participants were asked only to view the stories, and picture viewing times were recorded.

### Method

#### Participants

Based on the data collected from experiment 1, a power analysis was performed to determine the sample size needed for experiment 2. The analysis revealed that collecting data from 40 participants would result in high power (> .95). Thus, an additional 42 participants (27 female, mean age = 21.51, SD = 4.31) were sampled for experiment 2 from the same subject pool. Participants who completed experiment 1 were not allowed to participate in experiment 2.

#### Design

The same design used in experiment 1 was used in experiment 2, but the only dependent measure was picture viewing times.

#### Materials

The materials from experiment 1 were used again with one modification. We added one true or false comprehension question to the end of each filler story, for a total of 16 questions. Questions assessed participants’ understanding of broad themes and memory for explicit details presented in the texts and pictures (e.g., John’s meeting with Susan’s parents went well). All questions were specific to the individual filler stories participants had just read and were always presented after participants completed the final slide of the story. Half of the correct answers to the questions were true and the other half were false.

#### Procedure

The procedure for experiment 2 was identical to experiment 1 except with the exclusion of the unitization task and the inclusion of comprehension questions. Before beginning, participants were told that they would be asked to answer comprehension questions after some of the stories. Participants were also told that they would be asked to summarize their understanding of the overall narrative arc after reading the final story. This was done to ensure that readers were processing the stories, given the absence of the segmentation task. Participants proceeded through the materials using only the spacebar to advance to the next slide. For comprehension questions, the F and J keys were used to answer false and true, respectively. After reading all stories, participants then were asked to summarize their understanding of the overall narrative arc. These data were not analyzed further. Again, all stimuli, including the practice item, were presented using E-Prime version 2.0 (Psychology Software Tools, 2011).

#### Data cleaning

The same data cleaning procedure described in experiment 1 was used, resulting in 21 items being deleted from the analyses or 0.91% of the total data. No participant answered less than 75% of the comprehension questions correctly, indicating satisfactory comprehension of the narratives.

## Results and discussion

**Fig. 7:**
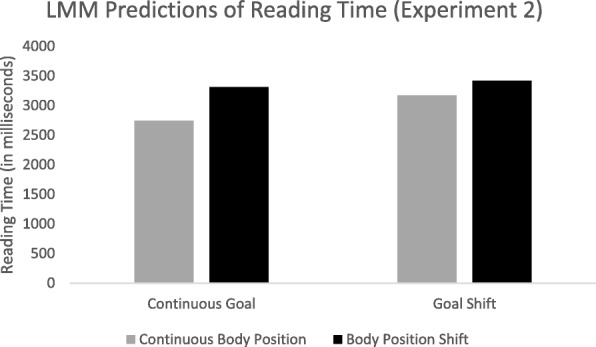
Predicted reading times for experiment 2 from linear mixed effects model by condition. LMM, linear mixed model

The dependent variable in experiment 2 was viewing time at the critical slide. This was again analyzed with linear mixed effect models in the lme4 package in R (Bates et al., 2015). Item and subject were treated as random in the null model. Both item (ICC = .24) and subject (ICC = .21) accounted for significant variance in viewing times. Next, the fixed effects of goal shift, change in body position, and the interaction between goal shift and change in body position were added. The model coefficients are presented in Table 6 and predicted reading times are presented in Fig. 7. The data suggest that shifts in goal and body position independently increase the time to view the critical slide (364 ms and 494 ms, respectively). This is consistent with the results of experiment 1 and suggests that both changes in goal and changes in body position affected the ease of processing for the critical slide, despite the fact that body position did not affect participants’ segmentation judgments in experiment 1.

**Table 6:** Model estimates for logistic linear mixed model predicting viewing time (experiment 2)

	Coefficient	SE	df	*t*	*p*
Fixed effects (log transformed)					
Intercept	2759	204	29.7	13.50	< .001
Goal	364	105	602.0	3.47	< .001
Body position	494	106	603.2	4.66	< .001
Goal × Body Position	− 240	148	602.0	−1.62	.106
Random effects					
Subject	362,246	602			
Item	442,438	665

### Story-wide analysis of situational features for viewing time

Parallel with experiment 1, we conducted an analysis assessing per-slide viewing times using our situational coding of the stories. As with experiment 1, we computed a linear mixed effects model predicting per-slide viewing times with causal shifts, spatiotemporal shifts, and body position shifts as fixed effects, and subject and story as random effects. We excluded the first slide of each story and viewing times longer than 3 SD from the mean (cutoff 8742 ms) or shorter than 500 ms were removed as outliers. The results of the analysis are in Table 7. As can be seen in Table 7, all three shifts were associated with an increase in reading time.^2^

**Table 7:** Results from linear mixed effects model using situational analysis variables (experiment 2)

	Coefficient	SE	df	*t*	*p*
Intercept	2971.00	142.36	71.05	20.49	<.001
Causal shift	436.91	30.95	7850.40	14.11	<.001
Spatial-temporal shift	153.38	43.00	7856.81	3.57	<.001
Body position shift	274.21	38.46	7854.54	7.13	<.001

These results demonstrate that body shifts are associated with longer viewing times, and that picture viewing times vary as a function of shifts in situational continuity, consistent with sentence reading-time experiments (e.g., Zwaan, Magliano, & Graesser, 1995). With respect to the purpose of experiment 2, these results indicated that changes in bodily position and goal discontinuities have an impact on processing time independent of the segmentation task.

## General discussion

Sequential visual narratives involve the cognitive coordination of linguistic and graphic content to convey a story (Cohn, 2016). Linguistic content can explicitly convey character goals and other internal states and visual content implies behavioral changes that are consistent or inconsistent with existing goals (Trabasso et al., 1989), and this information is used to explain character behaviors (Trabasso & Magliano, 1996; Trabasso & Suh, 1993). However, goals can also be inferred by changes in character behaviors (Long et al., 1992). We explored the role of these two sources of information on visual narrative processing. In experiment 1, participants made segmentation judgments and we found evidence that the continuity of goals via linguistic information affected these judgments at the target pictures, but changes in bodily position did not. Participants were more likely to perceive an event boundary at these pictures when the linguistic content indicated that there was a shift in the goals of the characters than when they implied continuity.

In contrast to the analyses for the critical slides, the supplemental analysis of the impact of situational changes and changes in bodily position on segmentation indicated that the latter had an impact on segmentation judgments in the larger story context. The critical panels reflect a unique situation in the context of the stories with respect to changes in body position and their relationships to explicitly established goals. Specifically, at the critical panels the actions were either consistent with an explicitly established goal or the goal interruption event, whereas, at other story locations the bodily change was not necessarily linked to an explicitly stated goal (when not linked, a new goal would presumably need to be inferred). As such, the data on the impact of bodily changes at the critical panel suggest that character actions are understood in the context of the explicitly established goals (which in this case were conveyed in language). If changes were consistent with an ongoing goal, then a boundary was less likely to be perceived than if it was consistent with a response to the interruption event. These data are consistent with those of Magliano and Zacks (2011) who showed that viewers of movies are less likely to perceive event boundaries when characters change locations when those changes in locations are consistent with prior character goals (i.e., there is a shift in the spatial-temporal framework, but no shift in character goals) than if they are not (i.e., there is a shift in both character goals and the spatial temporal framework).

However, across both experiments, we found evidence that changes in both goals and the bodily positions of characters lead to an increase in processing time in the critical panels. This was suggested by the supplemental analyses as well in that they indicated that changes in bodily position have an impact on processing times over and above the drastic changes in visual information that occurs when there are shifts in the spatial-temporal framework (see also Footnotes 1 and 2). These data are consistent with research on text comprehension indicating that understanders monitor continuity along multiple situational dimensions and that processing these changes increases processing effort (e.g., Zwaan, Magliano, & Graesser, 1995; Zwaan & Radvansky, 1998). The present study shows that viewers of visual narratives also monitor the characters’ bodily positions as they shift from picture/panel to picture/panel.

There are surprisingly few studies that have explored the extent to which picture processing times are sensitive to the processes that support mental model constructions in the context of visual narratives. For example, viewing times increase at pictures that require causal bridging inferences (Cohn & Wittenberg, 2015; Magliano, Kopp, Higgs, & Rapp, 2017; Magliano et al., 2016). Hutson, Magliano, and Loschky (2018) found that this increase was due to viewers producing additional eye fixations, indicating that viewers searched the pictures for information that supported the inference. The present study is the first study that we know of to show that viewing times of pictures in the context of sequential stories vary as a function of situational continuities along dimensions specified by the event indexing model (Zwaan & Radvansky, 1998). This raises an important question regarding how perceptual processes support recognizing that those shifts have occurred (see also Loschky, Hutson, Smith, Smith, & Magliano, 2018). While the present study was not conducted to address this issue, it illustrates the importance of understanding how perceptual processes support the comprehension of sequential visual narratives.

Why do viewing times increase when there are changes in bodily position and other situational factors? One potential answer comes from research on mental model construction in the context of text. It is well-documented that processing time is sensitive to model updating in the context of text (e.g., Kurby & Zacks, 2012; Zwaan, Langston, & Graesser, 1995). In research on text comprehension, increases in reading times are typically interpreted as reflecting the need to update the mental model in episodic memory (Zwaan & Radvansky, 1998). We assume that this is the case for visual narratives as well.

However, it may be the case that viewing times for pictures also reflects the need to update the representation of the scenes for visual narratives that are maintained in working memory (Loschky et al., 2018). Specifically, scene perception includes scene gist recognition (i.e., recognizing the basic category of a scene such as “street” or “interior”; Larson, Freeman, Ringer, & Loschky, 2014; Larson & Loschky, 2009; Loschky et al., 2007), object recognition (i.e., recognizing objects that exist within a scene; Davenport & Potter, 2004; Oliva & Torralba, 2007) and action recognition (Osaka, Matsuyoshi, Ikeda, & Osaka, 2010). Loschky et al. (2018) have argued that viewers of visual narratives must maintain a representation of the current scene in working memory across pictures/panels and establish how the scene representation for newly processed images are related to the prior representation in terms of gist, objects, and actions. The increase in processing time as a function of bodily position and changes in the spatial temporal framework may be reflective of updating the scene representation in working memory as well as updating the mental model in long-term memory. The present study was not designed to assess if working memory or long-term memory representations are being updated as a function changes in situational continuity and bodily position of character, but this is an important issue to address in subsequent research (see also Loschky et al., 2018). This is especially the case given that an inherent feature of comics is that there are missing actions between panels (McCloud, 1993), and understanding how viewers process missing actions is important to the study of the psychology of comics and visual narratives in general.

What has been learned about the multi-modal nature of processing sequential narratives? Like any multimodal information source, comprehenders must coordinate the visual and linguistic information to construct a mental model that accurately reflects the intended meaning of a narrative (Cohn, 2016; Magliano et al., 2013; Mayer, 2009). The current materials can best be characterized as co-dominant and co-assertive (Cohn, 2016), but they clearly carry different information by design. It is important to note that co-dominance reflects the idea that both streams carry unique information but does not necessarily mean that both the linguistic and visual content carry the same semantic weight (Cohn, 2016). One could argue that the materials used in this study contain linguistic content that conveys relatively more information about the narrative context than the visual content, in no small part because it specifies the narrative events, including the goals of the character. Assertiveness refers to the fact that there is a sequential structure and co-assertive means that both the visual and verbal content have a sequential structure. One test of this is to assess whether re-arranging the content disrupts its coherence. This is the case for both the text and the images used in the materials. However, this is not the case for the repeated pictures. As such, the verbal content is arguably more assertive in the context of these materials than the images. Nonetheless, the images at the critical target sentences would not make sense if they occurred earlier or later in the narrative. Clearly dominance and assertiveness may exist on a continuum and systematically exploring different kinds of relationships between these dimensions as delineated by Cohn (2016) would be important to understand the relative impact of linguistic and visual content on the processing of visual narratives.

Readers routinely infer how explicitly stated actions are causally connected to explicitly stated goals (Suh & Trabasso, 1993). This study was conducted with the assumption that viewers need to similarly infer how the actions of characters that are conveyed in images are similarly causally related to goals. The present study illustrates that changes in bodily position are monitored, but they do not signal a change in the event structure (i.e., signal an event boundary) when they are closely aligned with an explicitly stated and active goal in visual narratives. This is consistent with an interpretation that the actions reflected in the changes in bodily position are interpreted in the context of those goals. However, by no means does this study provide a definitive understanding of how viewers are able to understand the causal relationships between actions as depicted in pictures and goals as expressed in language. We hope that this study sparks interest in this issue, as understanding the relationship between goals and actions is an important source of coherence in narrative comprehension (Graesser, Singer, & Trabasso, 1994). Understanding how this is accomplished likely involves a coordination of processes that support scene perception and language processing that is unique to visual media (Loschky et al., 2018; Magliano et al., 2013).

It is important to discuss the ecological validity of our decision to repeat some images. While the convention of repeating images is not prevalent in graphic narratives, it is used in comic strips. The use in that context provided a justification for its use in this study. However, this situation could cause decreases in the assertiveness and dominance of the visual information stream. That is, because the images are identical across some of the panels, the impact of reordering them may be less disruptive to the meaning of the images relative to the verbal content. Additionally, because the critical stories contain at most four images, there is inherently less information that can be gleaned from them, thus causing a decrease in dominance relative to the verbal information. This possibility underscores the importance of testing a variety of visual narrative conditions to understand general principles that apply to understanding visual narratives.

This study fits into a growing body of research on visual narratives (e.g., Dunst, Laubrock, & Wildfeuer, 2018) and underscores the need for a theoretical framework to understand visual narratives (Bateman & Wildfeuer, 2014; Cohn, 2013, 2014; Loschky et al., 2018; Smith, 2012). To establish a coherent framework, researchers need to explore the visual, perceptual, and comprehension processes that occur during visual narrative understanding (Loschky et al., 2018). While much of the recent literature has focused on either text narratives (e.g., Kurby & Zacks, 2012; Zacks, Kumar, et al., 2009) or video with limited to no verbal content (e.g., Magliano & Zacks, 2011; Zacks et al., 2011; Zacks, Kumar, et al., 2009), sequential visual narratives (movies, comics, picture stories with language) offer researchers a flexible tool to explore how people understand real-world multi-modal media and how they coordinate these modalities to build mental representations (Cohn, 2016).

## Data Availability

The datasets used and analyzed during the current study are available from the corresponding author on reasonable request.

## Footnotes

1 It is possible that panels with different body positions may also differ in low-level perceptual qualities. We computed a pixel-to-pixel image similarity measure following the procedures used by Hard, Recchia, and Tversky (2011), Sherrill, Kurby, Lilly, and Magliano (2019) and Zacks, Kurby, Eisenberg, and Haroutunian (2011). For this procedure, we (1) scaled the images to 590 × 1050 to reduce the file size, (2) for each image, for each pixel, extracted the red, green, blue (RGB) values, (3) computed the 3-dimensional Euclidean distance in RGB values between each pixel and the corresponding pixel on the comparison (adjacent panel) image, (4) computed the mean pixel change per image. The body position change coding and the mean pixel change values were very highly correlated, *r* = .91. We asked whether body position changes predict changes in segmentation probability, and viewing time, after the effect of the low-level changes were factored out of the model. As such, we (1) computed a logistic mixed effects model, for the segmentation data, and a linear mixed effects model, for the viewing time data, using pixel change as a sole fixed factor, and subject and story as random effects, (2) extracted residuals from the model, and (3) predicted the residuals from the body position change coding. For both segmentation and viewing time, body position significantly predicted the residuals (segmentation: coefficient (coef) = .11, SE = .02, *t (17,190)* = 6.73, *p* < .001; viewing time: coef = 49, SE = 25, *t (16,593)* = 2.00, *p* = .046). This suggests that body position indicates change to the viewer beyond low level perceptual differences in the images.

2 A pixel-to-pixel change analysis, parallel to experiment 1, showed that body position change significantly predicted residuals (generated from a model predicting viewing time from pixel change), coef = 57, SE = 27, *t* (7900) = 2.14, *p* = .033.
